# NADPH diaphorase detects S-nitrosylated proteins in aldehyde-treated biological tissues

**DOI:** 10.1038/s41598-020-78107-6

**Published:** 2020-12-03

**Authors:** James M. Seckler, Jinshan Shen, Tristan H. J. Lewis, Mohammed A. Abdulameer, Khalequz Zaman, Lisa A. Palmer, James N. Bates, Michael W. Jenkins, Stephen J. Lewis

**Affiliations:** 1 Department of Pediatrics, Case Western Reserve University, Cleveland, OH 44106 USA; 2 Department of Pharmacology, University of Iowa, Iowa City, IA 52242 USA; 3 Department of Pharmacology and Physiology, University of Georgia, Athens, GA 30602 USA; 4 Department of Pediatrics, University of Virginia, Charlottesville, VA 801366 USA; 5 Department of Anesthesia, University of Iowa, Iowa City, IA 52242 USA; 6 Department of Bioengineering, Case Western Reserve University, Cleveland, OH 44106 USA; 7 Department of Pharmacology, Case Western Reserve University, Cleveland, OH 44106 USA

**Keywords:** Neurochemistry, Cellular imaging

## Abstract

NADPH diaphorase is used as a histochemical marker of nitric oxide synthase (NOS) in aldehyde-treated tissues. It is thought that the catalytic activity of NOS promotes NADPH-dependent reduction of nitro-blue tetrazolium (NBT) to diformazan. However, it has been argued that a proteinaceous factor other than NOS is responsible for producing diformazan in aldehyde-treated tissues. We propose this is a NO-containing factor such as an S-nitrosothiol and/or a dinitrosyl-iron (II) cysteine complex or nitrosated proteins including NOS. We now report that (1) S-nitrosothiols covalently modify both NBT and TNBT, but only change the reduction potential of NBT after modification, (2) addition of S-nitrosothiols or β- or α-NADPH to solutions of NBT did not elicit diformazan, (3) addition of S-nitrosothiols to solutions of NBT plus β- or α-NADPH elicited rapid formation of diformazan in the absence or presence of paraformaldehyde, (4) addition of S-nitrosothiols to solutions of NBT plus β- or α-NADP did not produce diformazan, (5) S-nitrosothiols did not promote NADPH-dependent reduction of tetra-nitro-blue tetrazolium (TNBT) in which all four phenolic rings are nitrated, (6) cytoplasmic vesicles in vascular endothelial cells known to stain for NADPH diaphorase were rich in S-nitrosothiols, and (7) procedures that accelerate decomposition of S-nitrosothiols, markedly reduced NADPH diaphorase staining in tissue sections subsequently subjected to paraformaldehyde fixation. Our results suggest that NADPH diaphorase in aldehyde-fixed tissues is not enzymatic but is due to the presence of NO-containing factors (free SNOs or nitrosated proteins such as NOS), which promote NADPH-dependent reduction of NBT to diformazan.

## Introduction

The similar spatial distribution of NADPH diaphorase and nitric oxide synthase (NOS) in aldehyde-treated tissues led to the postulation that the catalytic activity of NOS promotes NADPH-dependent reduction of nitro-blue tetrazolium (NBT) to insoluble readily visualized diformazan particles^1–3^. Since NADPH cannot directly reduce NBT^4,5^, it was argued that NBT binds to NOS at the flavin electron transport domain and is reduced by a NOS form, which was previously reduced by β-NADPH^1–3,6^. However, paraformaldehyde fixation of rat brain abolishes NOS activity in particulate and cytosolic fractions and although fixation abolishes NADPH diaphorase in the particulate fraction, 50–60% of NADPH diaphorase activity remains in the cytosolic fraction^7^. This suggests that cytosolic NADPH diaphorase in aldehyde-treated tissues results from a factor other than NOS. NOS contains heme iron, flavin adenine dinucleotide and flavin mononucleotide^8,9^. Hope and Vincent^1^ provided evidence that NADPH-dependent reduction of NBT in aldehyde-treated tissues is not directly due to NOS by demonstrating that metal chelators that inhibit activity of metalloenzymes and deflavinating agents did not eliminate NADPH diaphorase. While the NOS active site is not required to promote the NADPH-dependent reduction of NBT, some other reactive site of the protein could still be involved.

Chayen et al^10^ argued that NOS (or indeed any enzyme) cannot be directly responsible for diformazan production in aldehyde-treated tissues, and that an unidentified ‘proteinaceous material’ promotes NADPH-dependent reduction of NBT. Key points were that (1) α-NADPH is as effective as β-NADPH in promoting NBT reduction to diformazan^10^, (2) NOS activity is specific to β-NADPH^10^, (3) NOS activity is abolished by paraformaldehyde, and (4) NOS and NADPH diaphorase do not always show a similar distribution in tissues both under optical or electron microscopy scrutiny^11–13^. For example, Loesch et al^11,12^ found that NOS was associated with membranes of cytoplasmic vesicles in endothelial cells and nerve terminals whereas it was not in the lumen of these vesicles. In contrast, NADPH diaphorase was contained in the lumen but not membranes of the vesicles suggesting that a soluble factor generates NADPH diaphorase.

The presence of NOS on vesicle membranes would support the synthesis and storage of nitrosyl factors (NOFs) such as S-nitrosothiols (SNOs) and iron-nitrosyls and dinitrosyl-iron (II)-thiol complexes^14–19^. The lack of effect of metal chelators on NADPH diaphorase suggests that these NOFs are not iron-complexes. Interestingly, Hope and Vincent^1^ found that thiol-blockers markedly reduced NADPH diaphorase suggesting that free thiols or derivatives of free-thiols promote NADPH-dependent reduction of NBT. In the following work, we propose that the free-thiol derivative, NOFs, are responsible for the NADPH-dependent reduction of NBT. Indeed, there is now biochemical^20–24^ and functional^25–36^ evidence that many cell types including endothelial cells and neurons contain stores of NOFs although mechanisms of formation and storage are unknown.

The concepts driving this study are that (1) free small-molecular weight SNOs (e.g., S-nitrosocysteine and S-nitrosoglutathione)^19,37–42^ and NOS^43^ promote NADPH-dependent reduction of NBT in aldehyde-treated tissues, (2) SNOs transfer NO^+^ to (nitrosate) NBT allowing NADPH to directly donate electrons to NBT, and (3) NADPH diaphorase, in tissues and organelles such as cytoplasmic vesicles, is a histochemical marker for SNOs. The aim of this study was to provide direct evidence that NADPH diaphorase in aldehyde-treated tissues is due to preformed pools of SNOs that facilitate NADPH-dependent reduction of NBT to diformazan.

## Experimental procedures

### Ethical approval

All studies were carried out in accordance with the NIH Guide for the Care and Use of Laboratory Animals (Publication No. 80-23) revised in 1996. The animal use protocols were approved by the Animal Care and Use Committees of the University of Iowa and Case Western Reserve University.

### Spectrophotometer studies

Conversion of NBT or tetranitroblue tetrazolium (TNBT) to diformazan was monitored by a Beckman DV-70 spectrophotometer set initially at 580 nm, which is the optimal wavelength for authentic diformazan^4,5^. As will be seen, this wavelength was changed according to the exact reactants in the assay mixtures.

### Isolation of vesicles from endothelial cells

Sixty male Sprague–Dawley rats (14–16 weeks of age) were decapitated and small mesenteric arteries (150–250 μm internal diameter) were taken and placed in ice-cold Hepes-mannitol buffer (HMB) containing 5 mM Hepes, pH 7.4, 150 mM NaCl, 50 mM mannitol and 1 mM PMSF (phenyl-methyl-sulfonyl fluoride). Vesicles were extracted from endothelial cells as described by Moldovan et al^44^ with modifications. The vessels were inverted on a glass micropipette and the endothelium was carefully removed under a dissecting microscope and placed in HNB. The cells were centrifuged at 1,000* g* for 10 min and suspended in HMB without NaCl, at a ratio of 1 ml hypotonic lysis buffer to 3 mg of pellet, and lysed by gentle passing (15 times) through a Dounce homogenizer. The lysate (examined by phase-contrast microscopy) had organelles and intact nuclei. CaCl_2_ (final concentration of 10 mM) was added to the suspension, stirred for 20 min, and centrifuged at 5600*g* for 15 min. The pellet, containing nuclei, cytoskeletal proteins, and aggregated microsomes, was discarded and the supernatant was centrifuged at 100,000*g* for 10 min. The pelleted crude membrane fraction was resuspended in extraction buffer of 25 mM 2-morpholyno-ethanesulfonic acid buffered with NaOH at pH 6.5, 150 mM NaCl, 1 mM PMSF, and 1% triton X, for 20 min. Upon intermittent vortexing, the triton X-insoluble fraction recovered as a pellet after centrifugation at 12,000* g* (10 min at 22° C) was washed with MES buffer without triton X. This fraction was enriched in vesicles and contained no other organelles^44^.

### NADPH diaphorase histochemistry in rat tissues

Male Sprague–Dawley rats (300–350 g) were killed by decapitation and the brainstem-cerebellum and coeliac ganglia were removed and placed in ice-cold 0.1 M phosphate buffered saline (pH 7.5). Tissues were embedded in Histogel (Fisher Scientific) and frozen at − 20 °C for 10 min. The frozen blocks containing the tissues were sectioned into 10 µm thick slices on a cryostat (LEICA CM 1900, Vashaw Scientific). Sections were placed on Superfrost glass slides and allowed to dry at room temperature. The slides were then placed on ice and some sections were exposed to saline (0.9% w/v NaCl), 0.5 mM NaOH or 0.5 mM HCl for 5 min. Other sections were exposed to UV light (340–360 nm) for 30 min. The sections were thoroughly rinsed in ice-cold 0.1 M phosphate buffered saline (pH 7.5) and fixed in 0.4% paraformaldehyde for 2 h. NADPH diaphorase histochemistry consisted of incubating dried mounted sections with 0.1 M Tris buffer containing 0.3% Triton X-100, 0.1 mg/ml NBT, 1.2 mg/ml sodium malate, and 1.0 mg/ml -NADPH at room temperature in the dark for 60 min^45^.

### Mass spectrometry

Mass Spectrometry analysis was done using a Thermo Scientific TSQ Quantum Ultra AM triple stage quadrupole mass spectrometer equipped with a heated electrospray ionization (HESI) source in the positive ionization mode. The typical ion source parameters were as follows: spray voltage: 3000 V; sheath gas pressure (N_2_): 60 units; auxiliary gas pressure (N_2_): 20 units; ion transfer tube temperature: 275 °C; collision gas (Ar): 1.5 mTorr; Q1/Q3 peak resolution: 0.7 Da; and scan width: 0.002 Da. Samples were direct infusions (0.1 mM) of: NBT, TNBT, NBT + S-nitrosocysteine, or TNBT + S-nitrosocysteine, for 30 s and the data was collected in the mass range between 200 and 1000 m/z. Tandem mass spectrometry experiments were performed as above, but analyzing the 747 Da and 808 Da mass peaks with a collision energy of 40 eV. Experiments were performed in triplicate with no difference between the three samples when tested. All data was collected in centroid mode and were processed using Xcalibur 2.2 software (Thermo Fisher Scientific).

### Cyclic scanning voltammetry

All experiments were performed with 5-μm diameter parylene-insulated carbon fiber electrodes (CFE-2, ALA Scientific, Farmingdale, NY). We performed these experiments using an ITC-1600 (HEKA Corporation) voltage sources fed through a SR560 Stanford pre-amplifiers (Stanford Research), We alternated between 1 and − 1 V, with a scan rate of 50 mV/s, and a total experiment time of 5 min. The resulting data was notch filtered at 60 Hz and repeated runs were averaged together to produce the final curve.

### Synthesis of S-nitrosothiols

The SNOs, l-S-nitrosocysteine, l-S-nitrosoglutathione, dl-S-nitroso-homocysteine, dl-S-nitroso-N-acetylpenicillamine and l-S-nitrosopenicillamine were synthesized according to previously published methods^14,27–29^. All chemicals were purchased from Sigma (St. Louis, MO).

### Statistics

The data were analyzed by analysis of variance (ANOVA) followed by Student's modified *t* test with Bonferroni correction for multiple comparisons between means using error mean square values from the ANOVAs^46,47^. A value of *P* < 0.05 denotes statistical difference.

## Results

### Chemical modification of NBT and TNBT by S-nitrosocysteine

**Figure 1:**
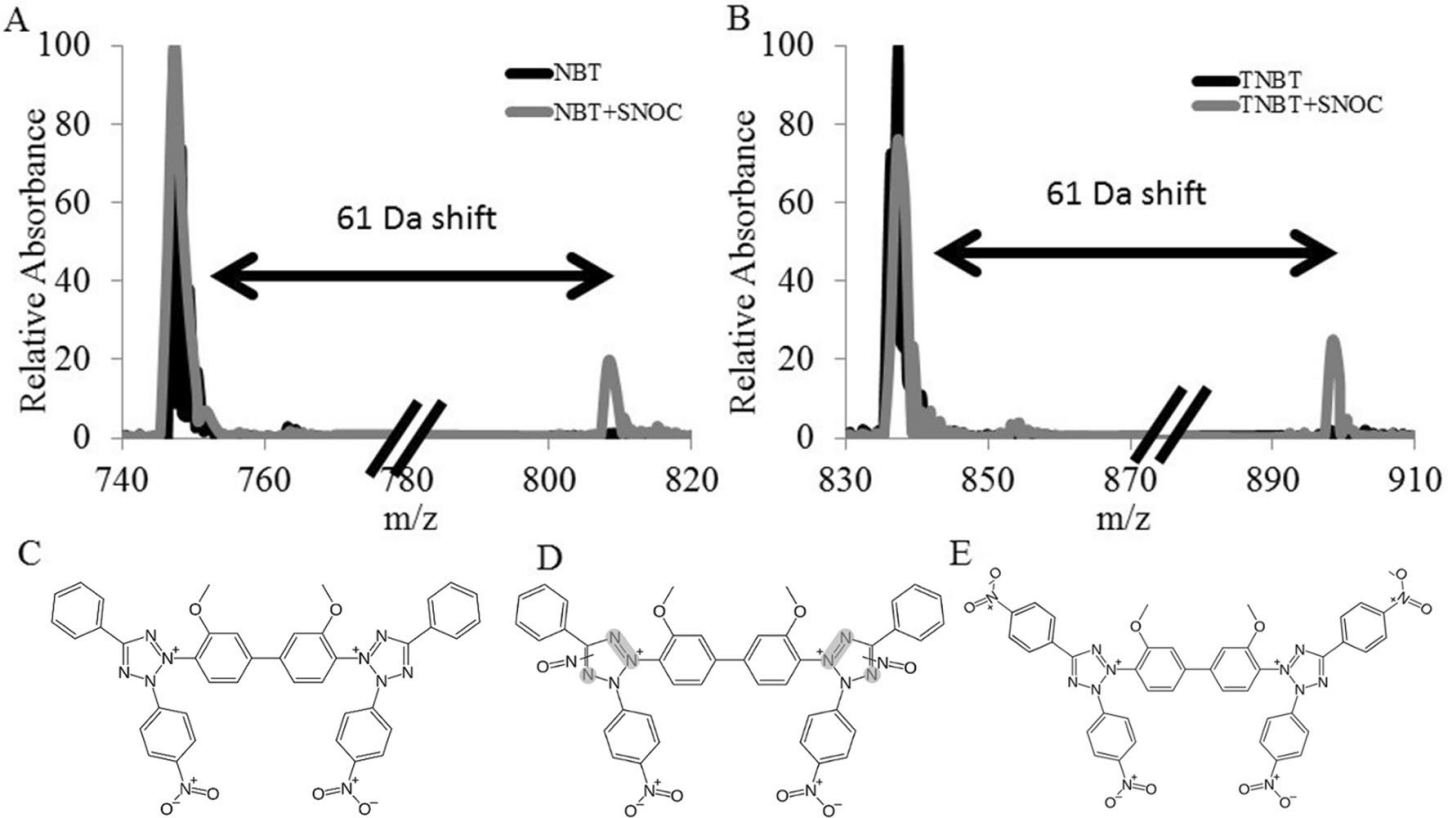
Mass Spectrum of NBT and TNBT in the presence of S-nitrosocysteine. Panels A and B. Mass spectrum of NBT (**A**) and TNBT (**B**) with (gray) and without (black) S-nitrosocysteine. The chemical structures for the NBT (**C**), NBT modified by reacting with a S-nitrosothiol (**D**), and TNBT (**E**).

**Figure 2:**
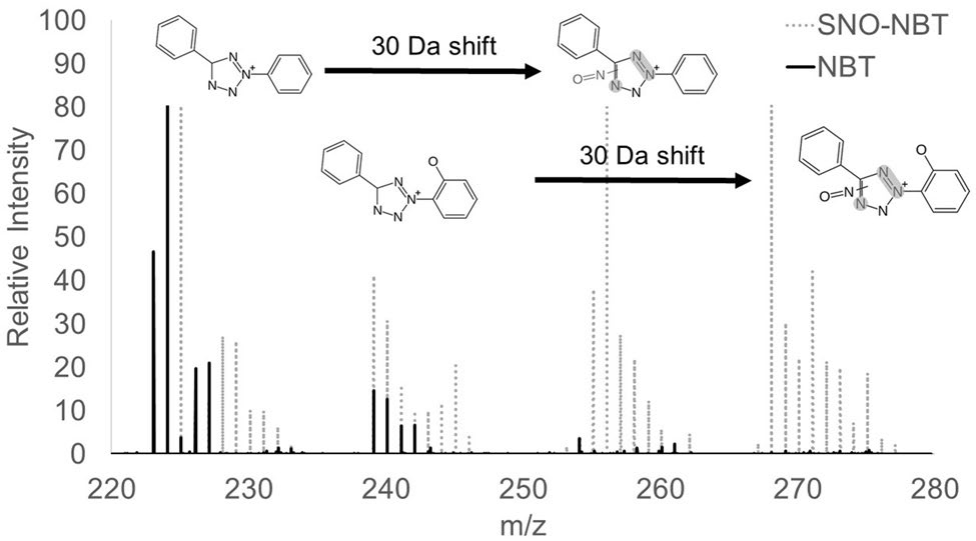
Tandem mass spectrometry results of either NBT (747 m/z, black) or SNO-NBT (808 m/z, grey). Striking differences can be seen in the range of 220 to 280 m/z which correspond to fragments of NBT which have been modified by the addition of an NO group.

**Figure 3:**
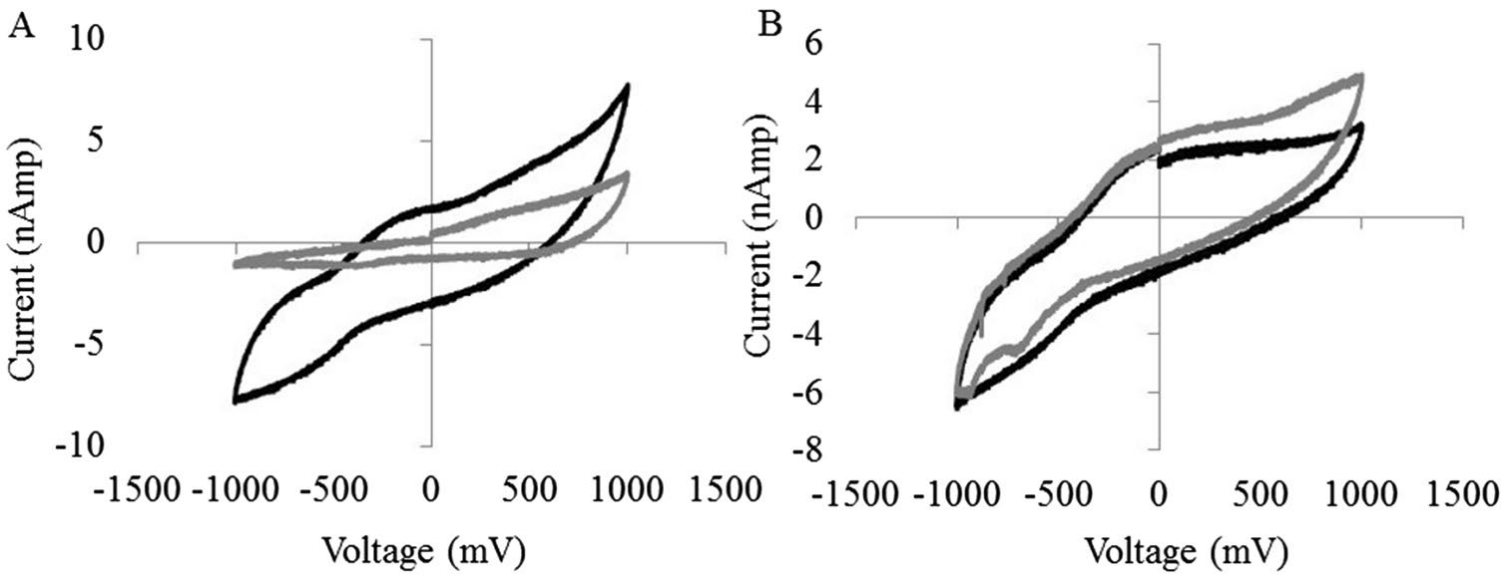
Cyclic scanning voltammetry Current versus Voltage graphs showing the oxidation and reduction peaks of NBT (**A**) and TNBT (**B**) with (gray) and without (black) S-nitrosocysteine.

We incubated 0.5 mM S-nitrosocysteine with 0.5 mM NBT or TNBT for 15 min in 10 mM Tris Buffered Saline, pH 7.4 with 4% formaldehyde. Without the presence of NADPH the NBT could not be reduced to diformazan and any chemical modifications made to NBT and TNBT by the S-nitrosothiol could be determined. We performed LC–MS on the samples after incubation and found that S-nitrosocysteine causes a 61 Da shift in both NBT and TNBT (Fig. 1A,B). This corresponds to addition of two NO groups and a single hydrogen atom. We also performed identical LC–MS experiments without S-nitrosocysteine and NADPH, and found no modifications. We then performed tandem mass spectrometry on the modified NBT molecule and ascertain it consisted of the addition of a NO group to each of the tetrazole groups in NBT (Fig. 2). We propose that the NO group is attaching to one of the nitrogen atoms of the tetrazole group (Fig. 2). This suggests that S-nitrosocysteine is equally capable of modifying both NBT and TNBT and poses the question of why it leads to the reduction of NBT by NADPH and not TNBT. To answer this, we employed cyclic scanning voltammetry to measure the oxidation and oxidation and reduction peaks of NBT and TNBT with and without S-nitrosocysteine. We found that S-nitrosocysteine had a marked effect on the oxidation and reduction peaks of NBT, but little effect on TNBT (Fig. 3A,B). While S-nitrosocysteine covalently modifies both molecules, it only changes the redox potential of NBT and facilitates its reduction by NADPH to diformazan.

### l-S-nitrosocysteine promotes NADPH-dependent reduction of NBT

**Figure 4:**
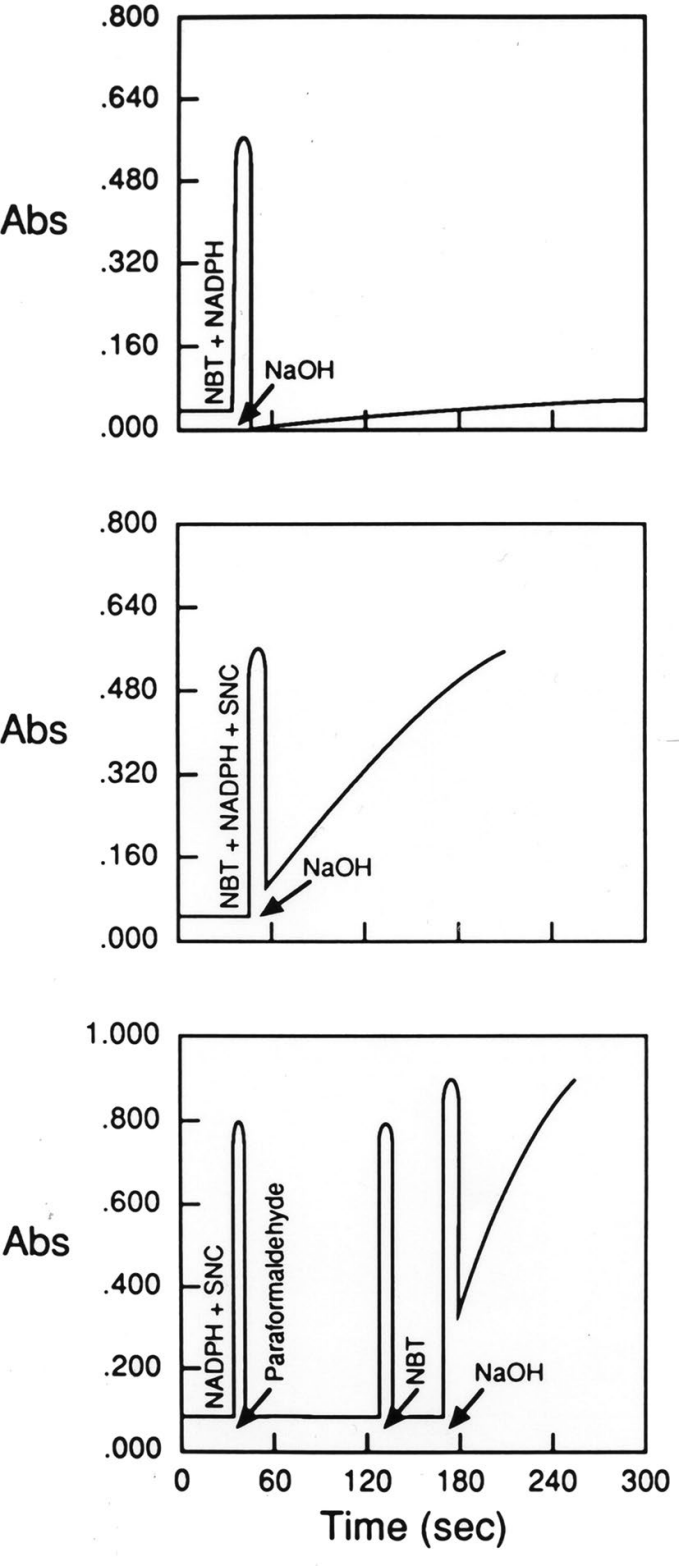
Typical examples of diformazan production produced by the addition of 1 M NaOH to solutions of NBT + β-NADPH (top panel), NBT + β-NADPH + S-nitrosocysteine (SNC) (middle panel), and β-NADPH + SNC) followed by paraformaldehyde (0.1%, final concentration), NBT and then NaOH (bottom panel). The final concentrations of these agents are those described in Table 1. Note that the initial spikes in absorbance are due to the opening of the cuvette chamber.

**Figure 5:**
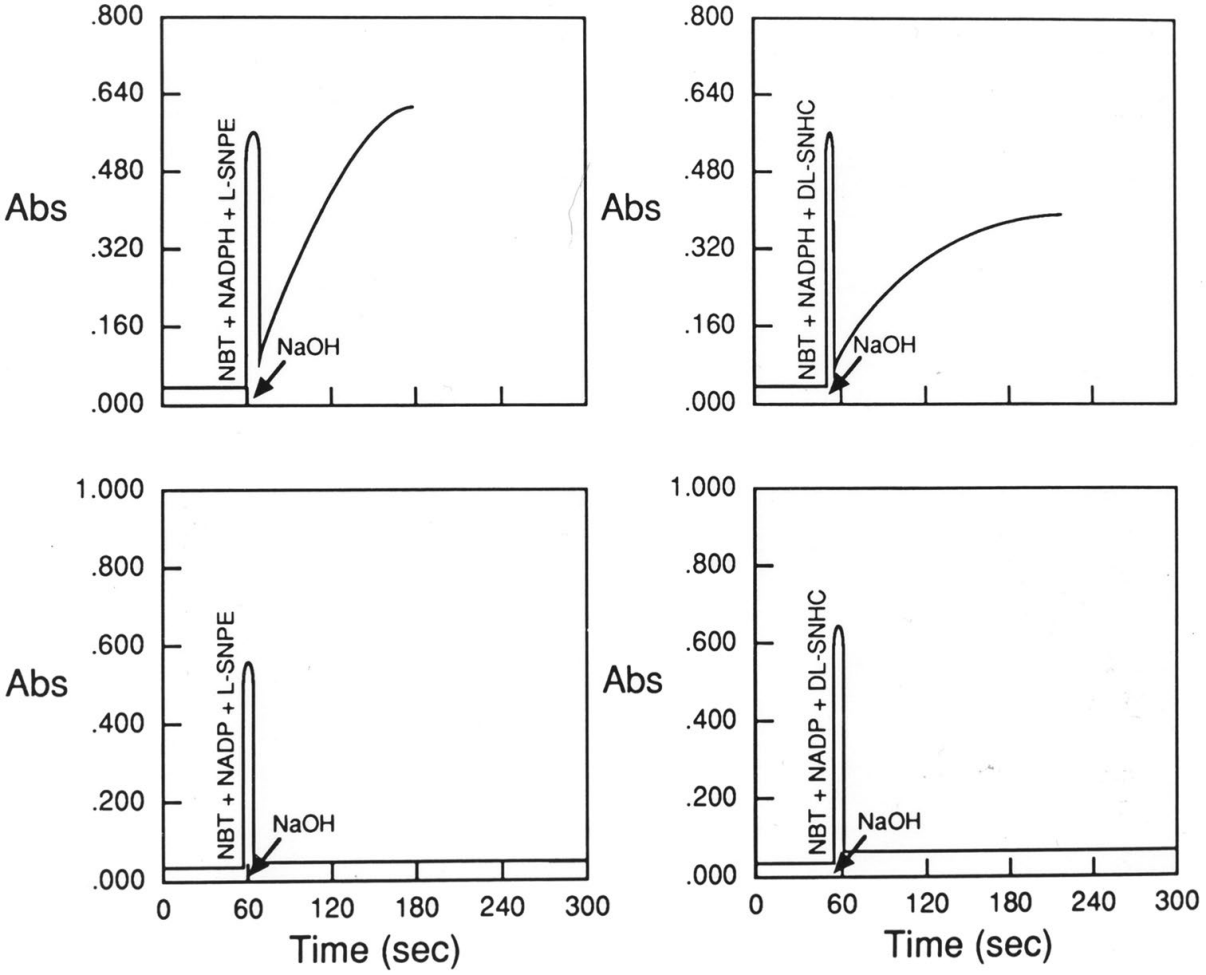
Typical examples of diformazan production produced by the addition of 1 M NaOH to solutions of NBT + β-NADPH + S-nitrosopenicillamine (L-SNPE) (top left panel), NBT + β-NADPH + S-nitrosohomocysteine (DL-SNHC) (top right panel), NBT + β-NADP + S-nitrosopenicillamine (L-SNPE) (bottom left panel) and NBT + β-NADP + S-nitrosohomocysteine (bottom right panel). The final concentrations of these agents are given in Table 1. Note that the initial spikes in absorbance are due to the opening of the cuvette chamber.

We added solutions of our test compounds to cuvettes and monitored the production of diformazan in a spectrophotometer (see Table 1). The spectrophotometer was initially set at 580 nm, which is the optimal wavelength for authentic diformazan^4,5^. However, our preliminary studies found that whereas the optimal wavelength for the optical detection of the diformazan by interaction of NBT with l-cysteine (see below) remained at 580 nm, the optimal wavelength for the optical detection of the diformazan elicited by interaction of NBT, NADPH and l-S-nitrosocysteine was 526 nm. This further suggests that l-S-nitrosocysteine modified (i.e., nitrosated) the NBT-diformazan moiety. Accordingly, the data in Figs. 4 and 5 are from experiments using the optimal wavelength for the detection of reaction product resulting each particular solution.

**Table 1:** Production of diformazan from nitroblue tetrazolium.

Solution number	Solution	Diformazan (absorbance units)
1	NBT	≈ 0
2	NBT + β-NADPH or NBT + α-NADPH	≈ 0
3	NBT + β-NADP	≈ 0
4	NBT + β-NADPH + paraformaldehyde	≈ 0
5	NBT + l-S-nitrosocysteine	0.03 ± 0.01
6	NBT + β-NADPH + l-S-nitrosocysteine	0.94 ± 0.05*
7	NBT + α-NADPH + l-S-nitrosocysteine	0.86 ± 0.06*
8	NBT + β-NADPH + paraformaldehyde + l-S-nitrosocysteine	1.19 ± 0.08*
9	NBT + β-NADP + l-S-nitrosocysteine	≈ 0
10	NBT + β-NADP + paraformaldehyde + l-S-nitrosocysteine	≈ 0
11	NBT + nitric oxide	0.06 ± 0.02
12	NBT + β-NADPH + nitric oxide	0.05 ± 0.02
13	NBT + β-NADPH + paraformaldehyde + nitric oxide	0.05 ± 0.02
14	NBT + cysteine	> 2.0
15	NBT + β -NADPH + l-cysteine	> 2.0
16	NBT + paraformaldehyde + l-cysteine	0.06 ± 0.03^†^
17	NBT + β-NADPH + paraformaldehyde + l-cysteine	0.04 ± 0.02^†^
18	NBT + l-cystine	≈ 0
19	NBT + β-NADPH + l-cystine	≈ 0
20	NBT + β-NADPH + paraformaldehyde + l-cystine	≈ 0

The times at which each of the compounds were added are depicted on the x-axis of Figs. 3 and 4. Conversion of NBT to diformazan was monitored by a Beckman DV-70 spectrophotometer. The optimal wavelength for diformazan produced upon addition of l-S-nitrosocysteine was at 526 nm. The optimal wavelength for diformazan produced upon addition of cysteine was 580 nm. The final concentrations of the S-nitrosothiols, NBT, NADPH and NADP were 0.5 mM. The final concentrations of cysteine, cystine and paraformaldehyde were 5 mM, 10 mM and 0.1%, respectively. Pure nitric oxide gas (i.e., completely devoid of oxygen) was bubbled directly into the described solutions for 20 min. The data presented are the mean ± SEM of absorbance units derived from 4–6 separate experiments.

*P < 0.05, absorbance values for solutions 6, 7 and 8 versus absorbance values for solutions 1–4, 5, 9–13, 16–20.

^†^*P* < 0.05, absorbance values for solutions 16 and 17 versus absorbance values for solutions 14 and 15.

It is known that β-NADPH will not directly reduce NBT^48^. Accordingly, we found that solutions of NBT, NBT + β-NADPH, NBT + β-NADP or NBT + β-NADPH + paraformaldehyde produced negligible amounts of diformazan over 60 min (solutions 1–4, Table 1). The addition of l-S-nitrosocysteine to NBT produced negligible diformazan (solution 5) demonstrating that l-S-nitrosocysteine does not readily reduce NBT. However, addition of l-S-nitrosocysteine to solutions of β-NADPH + NBT or α-NADPH + NBT resulted in the appearance of diformazan (solutions 6 and 7), which reached maximum within 25–30 min. Addition of l-S-nitrosocysteine to solutions of NBT + β-NADPH + paraformaldehyde resulted in similar amounts of diformazan (solution 8) as in solutions without paraformaldehyde, indicating that paraformaldehyde does not interfere with the ability of l-S-nitrosocysteine to promote the NADPH-dependent reduction of NBT.

Addition of l-S-nitrosocysteine to solutions of NBT + β-NADP did not produce diformazan in the absence or presence of paraformaldehyde (solutions 9, 10) suggesting that the electrons which reduce NBT in solutions of l-S-nitrosocysteine + β-NADPH + NBT originate from β-NADPH. This is important since β-NADP does not mimic β-NADPH in diformazan production in tissues^4,5,48^. Bubbling solutions of NBT with authentic NO in the absence of oxygen and hence NO_2_ for 20 min yielded only minor diformazan (solution 11). Bubbling solutions of NBT + β-NADPH or NBT + β-NADPH + paraformaldehyde with NO also yielded minor diformazan (solutions 12, 13). This suggests that NO does not promote NADPH-dependent reduction of NBT and that l-S-nitrosocysteine-induced facilitation of NADPH-dependent reduction of NBT is not due to decomposition of this S-nitrosothiol to NO. Reduced thiols such as cysteine will directly reduce NBT^49^. As expected, addition of NBT to solutions of l-cysteine or l-cysteine + β-NADPH resulted in immediate production of diformazan (solutions 14 and 15). It is known that paraformaldehyde covalently modifies free thiols which are then unable to reduce NBT^45^. Addition of NBT to solutions of paraformaldehyde + cysteine or paraformaldehyde + β-NADPH + cysteine did not yield diformazan (solutions 16 and 17). l-S-nitrosocysteine can decompose to NO and cystine^15^. However, addition of cystine to solutions of NBT, NBT + β-NADPH or NBT + β-NADPH + paraformaldehyde, did not yield diformazan (solutions 18–20).

### l-S-nitrosocysteine does not promote NADPH-dependent reduction of TNBT

NBT has two phenol and two *m*-nitrophenol substituents^4,5^. TNBT has instead four *p*-nitrophenol substituents, and resembles NBT in its UV–vis absorption spectrum and it redox potential^4,5^. As can be seen in Table 2, the mixture of TNBT, β-NADPH + l-S-nitrosocysteine yielded negligible amounts of diformazan. However, l-cysteine readily reduced TNBT to diformazan and as with NBT, paraformaldehyde prevented this reaction.

**Table 2:** Production of diformazan from tetranitroblue tetrazolium.

Solution number	Solution	Diformazan (absorbance units)
1	TNBT + β-NADPH	≈ 0
2	TNBT + l-S-nitrosocysteine	≈ 0
3	TNBT + β-NADPH	≈ 0
4	TNBT + β-NADPH + l-S-nitrosocysteine	0.04 ± 0.02
5	TNBT + cysteine	> 2.0*
6	TNBT + β-NADPH + l-cysteine	> 2.0*
7	TNBT + paraformaldehyde + l-cysteine	0.03 ± 0.02^†^
8	TNBT + β-NADPH + paraformaldehyde + l-cysteine	0.02 ± 0.02^†^

The times at which each of the compounds were added are depicted on the x-axis of Figs. 3 and 4. The final concentrations of l-S-nitrosocysteine, TNBT and β-NADPH were 0.5 mM. The final concentrations of cysteine and paraformaldehyde were 5 mM and 0.1%, respectively. The data are presented as mean ± SEM of absorbance units derived from 4–6 separate experiments.

*P < 0.05, absorbance values for solutions 5 and 6 versus absorbance values for all other solutions.

^†^*P* < 0.05, absorbance values for solutions 7 and 8 versus absorbance values for solutions 5 and 6.

### Effects of pH on the effects of S-nitrosothiols

The histochemical determination of NADPH diaphorase is optimal at pH 7.4–8.0^1^. In these studies, we added microliter amounts of 1 M NaOH to change the pH of the solutions from approximately 5.5 before addition of NaOH to pH 8.0 afterwards. Addition of NaOH to solutions of β-NADPH + NBT did not result in the production of diformazan (Fig. 4, top panel). In contrast, addition of NaOH to solutions of NBT + NADPH + S-nitrosothiols greatly accelerated the production of diformazan. As can be seen in the middle panel of Fig. 4, the addition of NaOH to solutions of NBT + β-NADPH +  l-S-nitrosocysteine, resulted in the rapid formation of diformazan which reached a maximum within 2–3 min. The initial spikes in absorbance are due to the opening of the cuvette chamber. The addition of NaOH to solutions of NBT + β-NADPH +  l-S-nitrosocysteine + paraformaldehyde, resulted in the more rapid formation of diformazan (Fig. 4, bottom panel). The final amounts of diformazan were similar in the absence or presence of paraformaldehyde. At present, we have no explanation for why the production of diformazan is faster in the presence of paraformaldehyde. Paraformaldehyde can form a formylcysteine moiety^50^, which might accelerate the reaction.

As expected, addition of NaOH to solutions of l-S-nitrosocysteine + β-NADP + NBT or S-nitrosocysteine + β-NADP + NBT + paraformaldehyde did not produce diformazan (data not shown). Addition of NaOH to solutions of either S-nitrosopenicillamine + β-NADPH + NBT or S-nitrosohomocysteine + β-NADPH + NBT resulted in the prompt formation of diformazan which reached maximum within 60 s (Fig. 5, top panels). In contrast, the addition of NaOH to solutions of these S-nitrosothiols and β-NADP + NBT, did not generate diformazan (Fig. 5, bottom panels). These findings demonstrate that S-nitrosothiols readily augment the NADPH-dependent reduction of NBT at a pH, which is optimal for diformazan production in tissues, and that this readily takes place in the presence of paraformaldehyde.

### NADPH diaphorase in tissues

The finding that overnight incubation of brain sections in buffers of pH 5–10 has little effect on NADPH diaphorase staining^1^ is not consistent with NOS-activity promoting NADPH-dependent reduction of NBT^8^. However, Hope and Vincent^1^ showed that NADPH diaphorase was markedly diminished in tissue sections that were incubated overnight in a buffer of pH 11. l-S-nitrosocysteine is stable at pH 3.0 and readily degrades to NO and cystine as pH is increased to pH 9.0. In contrast, S-nitrosoglutathione is relatively stable over pH of 3.0–9.0 but will rapidly degrade to NO at higher pH^51^. Therefore, elevations in the pH of aldehyde-treated tissues would lead to decomposition of S-nitrosothiols, if indeed these compounds are present in preformed stores. As such, elevating tissue pH prior to NADPH diaphorase histochemistry may reduce the concentrations of SNOs and therefore NADPH diaphorase.

We found that incubation of fixed brain sections with glycine–NaOH buffer (pH 9.1) substantially reduced subsequent NADPH diaphorase staining. However, we were concerned that such a long period of incubation at high pH may damage membranes and allow the translocation of diformazan particles. Indeed, we saw some particles of diformazan on the glass slides that had leached from the tissue. We then placed coronal sections from paraformaldehyde (4%)-perfused brain tissue on glass slides and exposed the sections to 0.5 mM NaOH or 0.5 mM HCl for 5 min. The slides were thoroughly rinsed with 0.1 M NaPO_4_ buffer (pH 7.4) and the tissues subjected to paraformaldehyde fixation and NADPH diaphorase histochemistry.

**Figure 6:**
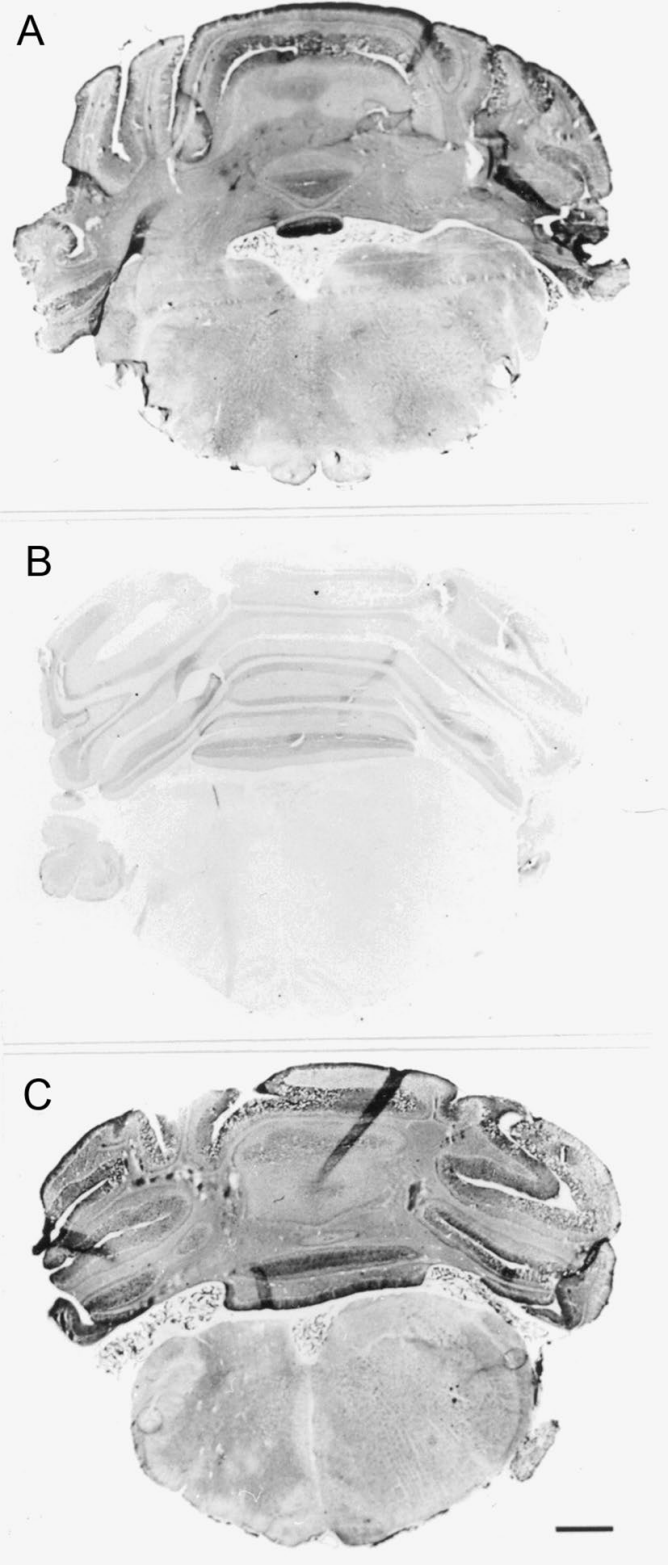
Examples of NADPH diaphorase staining in brain sections that were exposed to saline (0.9% w/v NaCl) (**A**), 5 mM NaOH (**B**) or 5 mM HCl (**C**) prior to conducting NADPH diaphorase staining. The bar at the bottom right hand corner represents 1 mm.

**Figure 7:**
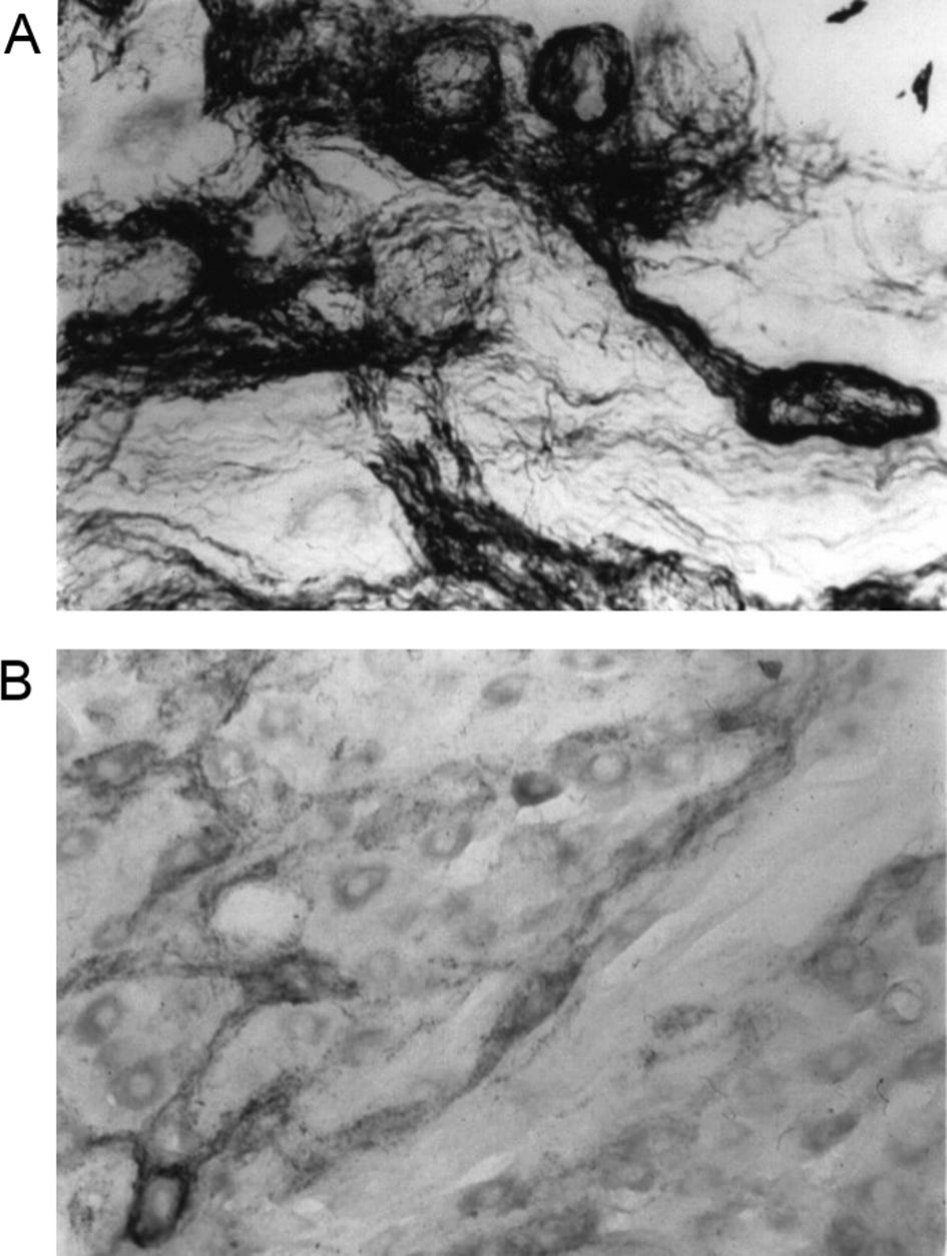
Examples of NADPH diaphorase staining in celiac ganglion sections that were exposed to room light (**A**) or UV light (**B**) for 15 min, prior to conducting NADPH diaphorase staining. The bar at the bottom right hand corner represents 50 μm.

A typical example of NADPH diaphorase staining is shown in Fig. 6 (top panel). Prior elevation of tissue pH virtually eliminated NADPH diaphorase (Fig. 6, middle panel) whereas a decrease in tissue pH failed to affect NADPH diaphorase (Fig. 6, bottom panel). There were no particles of diformazan that had dissociated from the tissue sections. These findings suggest that alkalization of tissues may abolish NADPH diaphorase by the pH-dependent decomposition of S-nitrosothiols. S-nitrosothiols such as S-nitrosocysteine rapidly degrade upon exposure to UV light^51^. We exposed celiac ganglion sections (5 μm) from rats to UV or room light for 15 min. The sections were incubated for 2 h in 0.1 M phosphate-buffered 4% paraformaldehyde (pH 7.4) and then subjected to NADPH diaphorase histochemistry. Panel A of Fig. 7 shows NADPH diaphorase in the preganglionic terminals in a section that was exposed to room light. These terminals are wrapped around post-ganglionic cell bodies that do not stain for NADPH diaphorase. Panel B of Fig. 7 shows that NADPH diaphorase staining in preganglionic terminals was markedly diminished by prior exposure of the tissue sections to UV light.

### S-nitrosothiols in cytoplasmic vesicles of endothelial cells

**Figure 8:**
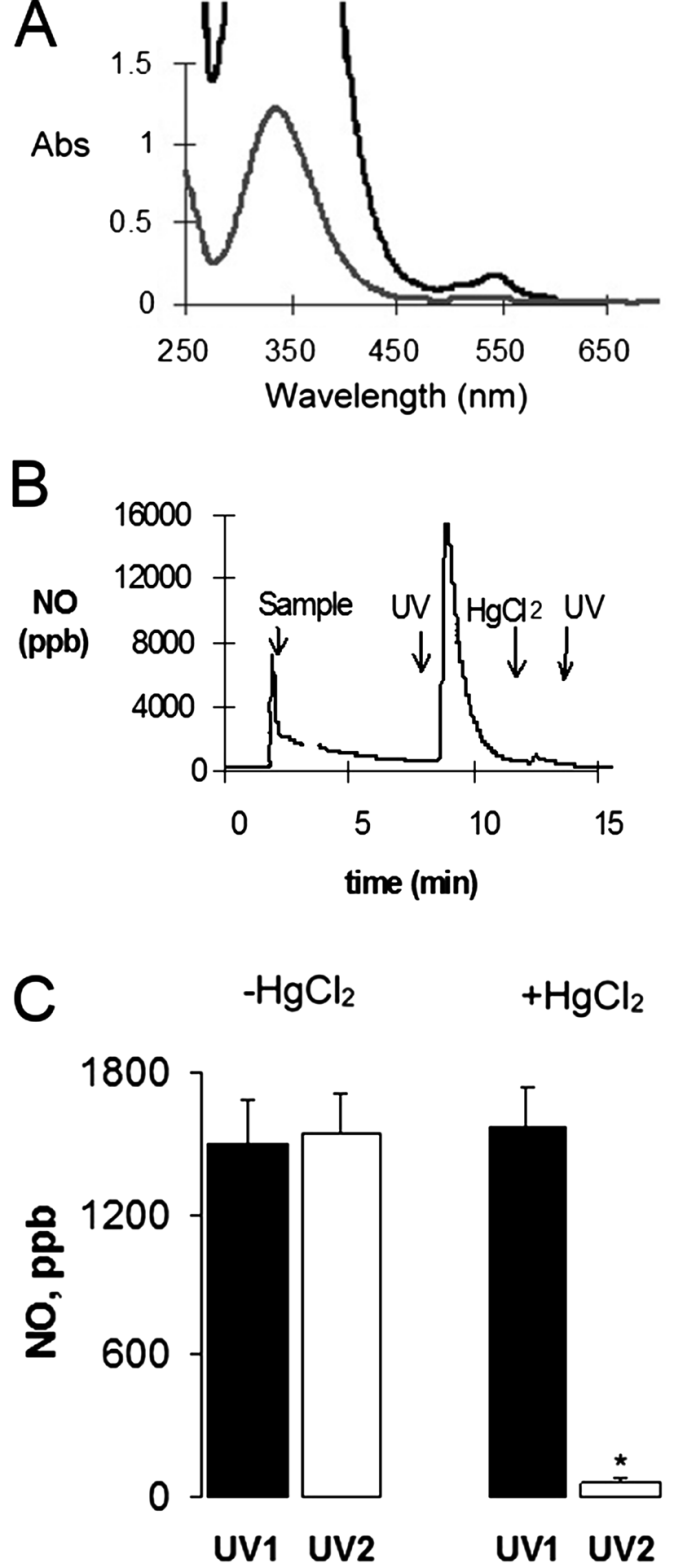
Direct detection of S-nitrosothiols in endothelial vesicles. (**A**) Vesicle membranes were lysed by sonication and the luminal contents and were examined spectrophotometrically. The lower spectrum is the same sample as the upper spectrum diluted seven-fold in water. (**B**) Typical example of NO-release from vesicle contents by ultra-violet (UV) light before and after addition of HgCl_2_. (**C**) Summary of NO-release from vesicle contents by ultra-violet (UV) light before and after addition of HgCl_2_. The data are mean ± SEM of 5 separate experiments. *P < 0.05, NO release upon exposure to UV light after addition of HgCl_2_ (UV2) as compared to before addition of HgCl_2_ (UV1). HgCl_2_ exposure abolishes NO release from vesicles.

We collected vesicles from endothelial cells harvested from small rat mesenteric arteries, lysed the vesicle membranes by sonication, and examined the luminal contents spectrophotometrically. The absorption spectra of the luminal contents are shown in Fig. 8A. The lower spectrum is the same sample as the upper spectrum diluted seven-fold in water. The spectra were characteristic of S-nitrosothiols with a large absorption peak at 340 nm and a smaller peak at 545–550 nm^14,51^. The sonication of these vesicles resulted in an estimated concentration of SNOs of 1.3 ± 0.2 mM (based on absorption-concentration curves of S-nitrosocyteine, the major identified endothelium-derived SNO^14^). In other studies, the vesicles were disrupted by sonication and the luminal contents were examined in a chamber in which the released NO was continuously measured by drawing a stream of nitrogen through the headspace into a chemiluminescence NO detector (Dasibi, model2108; Glendale, CA)^14^. The results of a typical experiment are shown in Fig. 8B. The initial NO peak upon addition of the sample to the chamber, represents NO previously formed in the solution. Subsequent irradiation of the solution with long-wave UV light (340–360 nm) for 15 s caused a large volume of NO to be released. This is a classic sign that S-nitrosothiols were present in the solution^14,51^. The UV light-induced release of NO could be repeated many times as the decomposition of SNOs by each brief exposure to UV light represented only a small portion of the SNOs in solution. When the samples were treated with mercuric chloride (HgCl_2_, 0.05 M), which causes decomposition of SNOs with release of inorganic nitrite^14,51^, no further NO could be generated by exposure to UV light. The findings of these studies are summarized in Fig. 8C. As can be seen, two episodes of UV light elicited similar bursts of NO in the absence of HgCl_2_. In contrast, the second burst of UV light elicited minimal NO when applied after the addition of HgCl_2_.

## Discussion

This study addresses the process(es) that lead to the staining generally ascribed to NADH diaphorase activity in aldehyde-treated tissues. Despite the proposition that NADPH diaphorase results from the catalytic activity of NOS^1–3^ this proposition seems untenable for numerous reasons put forward by Chayen et al^10^ and as summarized in Table 3. The possibility that NADPH diaphorase is due to proteins other than NOS derives from the fact that functionally NOS is a combined P450 and P450 reductase complex in a single protein^8^. Since the initial hypothesis was that NBT binds to NOS at the flavin electron transport domain of the cytochrome P450 reductase region and steals the electrons that are donated to the enzyme by β-NADPH^1–3^, the unanswered question must be why NADPH diaphorase does not also stem from free cytochrome P450 reductase that is not associated with NOS^52,53^.

**Table 3:** Arguments against NADPH diaphorase being NO synthase.

Arguments and citations
**1. NADPH-dependent reduction of NBT in aldehyde-treated tissues is not due to NOS activity**
NOS is a P_450_ enzyme, which contains heme iron and flavins. Metal chelators and deflavinating agents that eliminate NOS activity do not eliminate NADPH diaphorase histochemistry^1,7^
**2. NOS and Cytochrome P450 reductase should both contribute to NADPH diaphorase**
The hypothesis that NBT binds to NOS at the flavin electron transport domain of the cytochrome P450 reductase subunit and steals the electrons that are donated to the enzyme by β-NADPH must also pertain to free cytochrome P450 reductase molecules that are not associated with NOS^1–3,7,47,48^
**3. α-NADPH drives the NADPH diaphorase reaction but not NOS activity**
α-NADPH is as effective as β-NADPH in promoting the reduction of NBT to diformazan. Since α-NADPH will not donate electrons to NOS, it is unlikely that diformazan is due increase in the catalytic activity of NOS, especially in light of evidence that NOS activity is abolished in paraformaldehyde-treated tissue^6,8^
**4. NOS activity is absent in paraformaldehyde-treated tissues – NADPH diaphorase remains**
Fixation of rat brain with 4% paraformaldehyde abolished NOS activity in particulate and cytosolic fractions. Although fixation abolished NADPH diaphorase in the particulate fraction, 50–60% of NADPH diaphorase activity remained in the cytosolic fraction^6^
**5. A soluble factor in cytoplasmic vesicles is responsible for generation of NADPH diaphorase**
NOS and NADPH diaphorase do not always show the same distribution in tissues at light or ultrastructural levels. For example, NOS is found in membranes of cytoplasmic vesicles in endothelial cells and nerve terminals but not in the lumen of these vesicles. In contrast, NADPH diaphorase is found in the lumen but not membranes of the vesicles^9–11^
**6. S-nitrosothiols generate NADPH diaphorase**
The present study demonstrates that S-nitrosothiols initiate the NADPH-dependent generation of diformazan from NBT but not TNBT in the absence or presence of paraformaldehyde

The present studies provide compelling evidence that (1) SNOs exist in cytoplasmic vesicles of endothelial cells of rat small mesenteric arteries, (2) SNOs promote the NADPH-dependent reduction of NBT, (3) both α-NADPH and β-NADPH promote the reduction of NBT to diformazan and since α-NADPH will not donate electrons to NOS^10^, it is unlikely that diformazan is the result of an increase in the catalytic activity of NOS, (3) prior depletion of SNOs in tissues markedly reduces subsequent NADPH diaphorase staining. In contrast, NO caused only minimal diformazan production, or more precisely, minimal change in absorbance, not further augmented by NADPH with or without paraformaldehyde. The maximum absorbance of authentic diformazan is 580 nm^4,5^. However, the maximum absorbance of the diformazan generated by addition of SNOs to solutions of NBT and β-NADPH was found to be 526 nm. Importantly, Schmidt et al^54^ demonstrated that the absorption band maximum of diformazan extracted from brain tissue was 520 nm. The studies with TNBT show that reaction with SNOs produces similar structural changes in TNBT as in NBT, at least to the extent that there are corresponding 61 dalton mass shifts seen in mass spectrometry. This is suggestive of a nitrosation reaction and it appears from the fragments it is seen in to be associated with the tetrazole ring. Yet while this produces substantial changes in the redox chemistry of NBT as seen by cyclic voltammetry, it produces at most only minor changes in the redox properties of TNBT. Reaction with SNOs imparts on NBT the ability to be reduced directly by NADPH, it does not do so on TNBT. How the change in redox potential is related to the increased reactivity with NADPH cannot be determined without a better understanding of the chemistry involved, but they do appear to be correlated.

It is important to note that despite similar physicochemical properties, histochemical distribution of NADPH diaphorase is widely different when NBT or TNBT are used in the assay mixture. NBT produces a much greater presence and wider subcellular distribution such as presence in cytoplasmic vesicles^55–58^. It could be that the presence of NADPH diaphorase from NBT in aldehyde-treated tissues may represent nitrosated proteins whereas the NADPH diaphorase from TNBT may strictly pertain to individual (non-enzymatic) detection of specific proteins such as peroxidase and alkaline phosphatase^55,56^. Taken together, the above findings support that SNOs nitrosate NBT, altering its redox potential, and facilitate the direct transfer of electrons from NADPH.

Our evidence that the ability of l-S-nitrosocysteine to promote NADPH-dependent reduction of NBT readily occurred in the presence of paraformaldehyde also suggests that NADPH diaphorase in aldehyde-treated tissues actually defines the presence of S-nitrosothiols. Moreover, since the SNO-mediated NADPH-dependent reduction of NBT is non-enzymatic, it would be more appropriate to refer to this process as the production of diformazan rather than to the NADPH diaphorase activity of SNOs. Addition of NaOH to solutions of NBT + β-NADPH +  l-S-nitrosocysteine resulted in the more rapid formation of diformazan in the presence of paraformaldehyde (Fig. 1 Middle/Bottom), although the final amounts of diformazan were similar in the absence or presence of paraformaldehyde. At present, we have no direct explanation for why the production of diformazan is faster in the presence of paraformaldehyde. However, it is known that paraformaldehyde can form a formylcysteine moiety^50^, which might accelerate the transfer of NO or NO^+^ from S-nitrosothiols. NOS contains functionally critical iron and flavin moieties^8^. Therefore, existing evidence that metal chelators (known to inhibit the activity of metalloenzymes) and deflavinating agents that would eliminate NOS activity do not reduce NADPH diaphorase staining^1^ suggests that NADPH diaphorase is not due to the catalytic activity of NOS or the presence of iron-nitrosyls or dinitrosyl-iron (II)-thiol complexes. Evidence that thiol-chelators markedly reduce NADPH diaphorase supports the concept that free thiols are converted to S-nitrosothiols such as l-S-nitrosocysteine, which in turn promote NADPH-dependent reduction of NBT.

It is important to note that the term “NADPH diaphorase” is meant to strictly denote the β-NADPH-driven enzymatic reduction of NBT to diformazan via active enzymatic processes. As would be expected, fixation of biological tissues and particular the degree of inhibition of enzyme activity is both concentration- and time-dependent^59,60^. Although paraformaldehyde fixation can eliminate the activity of certain enzymes such as NOS^7^, alcohol dehydrogenase and glutamate dehydrogenase^61^, it does not necessarily eliminate all enzymatic activity in tissues. For example, Janigan et al^60^ that complete fixation with paraformaldehyde only inhibited the activities of various phosphatases by about 50% whereas Seligman et al.^59^ found that fixation of rat liver with a 10% solution of paraformaldehyde (formalin) for 24 h preserved between 26 and 87% of activity of a wide variety of enzymes. β-glucuronidase was the most resistant to the inactivating effects of formalin. At a time when fixation was satisfactory for preparing frozen sections (24 h), considerable enzymatic activity was present in decreasing order for β-glucuronidase, sulfatase, acid phosphatase, esterase, and alkaline phosphatase. At present, we do not know whether the SNO-initiated NADPH-dependent generation of NADPH diaphorase is enzyme-mediated but suggest that if an enzyme is involved it does not rely on the β-NADPH-dependent electron transfer to proteins for its activity since we have found that α-NADPH is as effective as β-NADPH for driving the NADPH diaphorase reaction.

In summary, the present study provides compelling evidence that NADPH diaphorase in aldehyde-treated tissues may arise from the ability of endogenous SNOs such as S-nitrosocysteine and S-nitrosoglutathione to promote NADPH-dependent reduction of NBT. This is most likely to be due to the SNO-mediated nitrosylation of NBT, which is then able to directly accept electrons from α- or β-NADPH. As such, this new view of NADPH diaphorase as defining the presence of SNOs and nitrosylated proteins including NOS itself^62–65^ can be seen as a strong redefinition of the current concept that NADPH diaphorase defines the tissue distribution of NOS. In particular, we suggest that NADPH diaphorase is an invaluable tool for the visualization of S-nitrosylated proteins and preformed pools of SNOs in biological tissues. Future studies designed to address the relationships between the distribution/localization of NADPH diaphorase and those of neuronal, endothelial and inducible forms of NOS in tissues such as the brain^66^ and sensory cells^67^ would build on the new concept of the biological significance of NADPH dipahorase. These studies could be done in healthy and disease tissue using selective inhibitors of the NOS isozymes enzymes^68^ to establish the active roles of NOS isoforms in producing nitrosated proteins/free SNOs.

### Significance

NADPH diaphorase has been widely used and accepted as a histochemical marker for the presence of NOS in aldehyde-treated tissues at the light and ultra-structural level. Here we present data, which refutes this established view-point, and open the way for a new interpretation of existing data. We propose that NADPH diaphorase is not a marker for NOS, but rather as a marker for NOS products including NO-containing factors such as free SNOs, nitrosated proteins including NOS, and dinitrosyl-iron (II) cysteine complexes.
